# A huge lacrimal gland ductule dacryolith with a hairy nucleus: a case report

**DOI:** 10.1186/s12886-018-0915-y

**Published:** 2018-09-12

**Authors:** Jiao Zhao, Zhike Xu, Aijun Han, Li Zeng, Gengsheng Hao, Bin Chen

**Affiliations:** 1 Departments of Ophthalmology, The People’s Hospital of Leshan, Leshan, Sichuan People’s Republic of China; 2 Department of Pathology, The People’s Hospital of Leshan, Leshan, Sichuan People’s Republic of China

**Keywords:** Lacrimal gland ductule, Dacryolith, Conjunctivitis

## Abstract

**Background:**

Dacryoliths in lacrimal gland ductule are a rare condition and an unusual cause of conjunctivitis. Here we report a case with a large lacrimal gland ductule stone with an unique hairy nucleus.

**Case presentation:**

A 38-year-old female presented with a red left eye for one month. Physical examination revealed an inflammatory granuloma in the lateral canthus and a fistula with purulent secretion. Antibiotics did not ameliorate the symptoms. After we incised the fistula, a huge dacryolith (10 mm × 5 mm × 3 mm) was identified at the end of the dilated lacrimal gland ductule and was removed surgically. Histopathologic examination showed a hairy nucleus surrounded by lamellar structure. The symptoms were resolved in 2 weeks after dacryolith extraction. The formation of the hair-centered dacryolith might be associated with the patient’s personal history of being a rabbit raiser for years.

**Conclusion:**

We should be aware of lacrimal gland ductule dacryolith as a rare cause of conjunctivitis. The relationship between dacryolithiasis and fur-bearing animal raising warrants further investigation.

## Background

Conjunctival congestion is commonly caused by infection, foreign bodies, and autoimmune disorders [1]. Unusually, conjunctival redness could be due to lacrimal gland ductule dacryolith. Stones in lacrimal gland ductule are a rare condition [1–5]. Here we describe a case with a large lacrimal gland ductule dacryolith with a unique hair nucleus.

## Case presentation

**Fig. 1:**
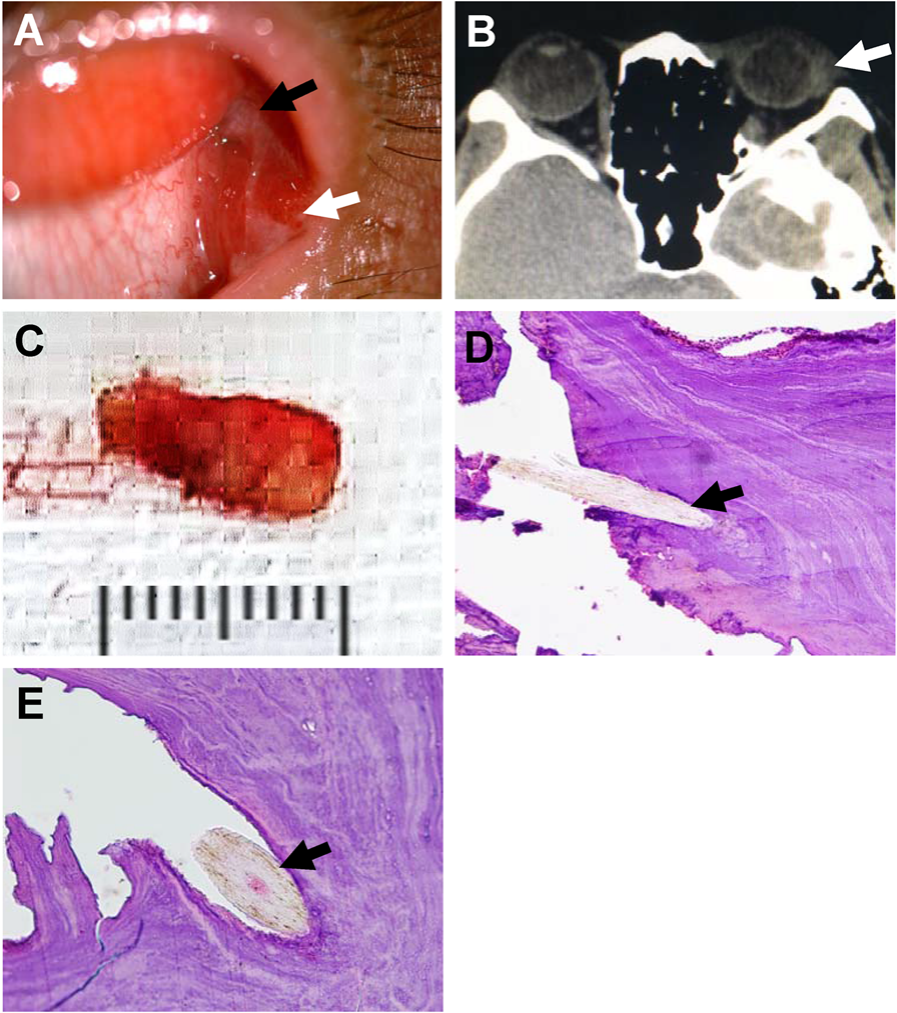
**a** Prolapsed lacrimal gland (black arrow) and an inflammatory granuloma with a fistula (white arrow) with purulent secretion. **b** A computerized tomography scan showing swelling of the left lacrimal gland (white arrow). **c** The complete dacryolith extracted from the dilated lacrimal gland ductule. **d**, **e** Histopathologic examination of the dacryolith that has a hairy nucleus (black arrow) with hematoxylin and eosin staining. Magnification: 20X for (**d**) and 40X for (**e**)

A 38-year-old female presented with a red left eye for one month. Physical examination revealed an inflammatory granuloma in the lateral canthus and a fistula with purulent secretion. Lateral conjunctival congestion and chemosis were observed (Fig. 1a). A computerized tomography scan detected swelling in the area of the left lacrimal gland (Fig. 1b). Under topical and local anesthesia, a blunt needle was inserted into the fistula, and then the fistula was incised. A huge dacryolith (10 mm × 5 mm × 3 mm) was identified at the end of the dilated lacrimal gland ductule and removed surgically (Fig. 1c). Histopathologic examination showed a hair nucleus surrounded by lamellar structure (Fig. 1d, e). The symptoms were resolved in 2 weeks after dacryolith extraction. Further history taking revealed that the patient had raised hundreds of rabbits in an enclosed room since she was 10 years old and that she had experienced foreign body sensation for many years, suggesting that the hairy nucleus might be a rabbit fur.

## Discussion

The pathogenesis of lacrimal gland ductule dacryolith formation is still largely unknown. Previous studies demonstrated that stones may develop from a nucleus of various components. Baratz suggested that an eyelash could be a nidus for dacryolith formation in the lacrimal gland [2]. Epithelial debris and foreign hairs might also be the initiating agent in the dacryolith formation. Baker and Bartley reported two cases with lacrimal gland ductule stone, and histopathologic examination disclosed an obvious nidus of unknown material in the center of the dacryolith [1]. Halborg et al. reported 3 cases of lacrimal gland stones which were composed of amorphous material organized in lamella and separated by a granular-transitional zone [3]. Alten et al. presented a series of 4 cases with dacryolith, one of which contained amorphous acellular organic material with a cilium surrounded by inflammatory cells [4]. Embedded cilia was also found in another case with lacrimal ductule dacryolith [5]. Based on this patient’s life experience and the results of histopathologic examination, the hairy nucleus of the dacryolith might be either a cilium or a rabbit fur.

Alteration in tear fluid dynamics contributes to dacryolithogenesis as well. Tear stasis leads to an accumulation of debris and proteins, which represents the nidus of the stone [4]. Therefore, the hair nucleus-initiated stone formation may be accelerated by tear stasis.

Patients with lacrimal gland ductule dacryolith could be asymptomatic or present with conjunctivitis when associated with infection. The diagnosis could be easily missed [6]. Physicians should consider lacrimal gland ductule dacryolith in differential diagnosis of chronic conjunctivitis which poorly responds to medical treatment.

## Conclusion

Physicians should be aware of lacrimal gland ductule dacryolith as a rare cause of conjunctivitis. The relationship between dacryolithiasis and fur-bearing animal raising warrants further investigation.
